# Postoperative Pain Management in DIEP Flap Breast Reconstruction: Identification of Patients With Poor Pain Control

**Published:** 2010-09-15

**Authors:** Eran D. Bar-Meir, Janet H. Yueh, Philip E. Hess, Christoph E. A. Hartmann, Munique Maia, Adam M. Tobias, Bernard T. Lee

**Affiliations:** ^a^Department of Surgery, Division of Plastic and Reconstructive Surgery; ^b^Department of Anesthesiology and Critical Care, Beth Israel Deaconess Medical Center, Harvard Medical School, Boston, MA

## Abstract

**Objective:** Adequate control of postoperative pain directly improves patient satisfaction and outcomes, and timely identification of patients with poorly controlled pain is essential. Pain management protocols are best studied in patients recovering from the same operation. In our institution, the postoperative pain regimen for patients undergoing deep inferior epigastric perforator (DIEP) flap breast reconstruction is standardized using patient-controlled analgesia (PCA) followed by conversion to oral narcotics. From this uniform population, we were able to identify a subgroup of patients with poor pain control. **Methods:** Over a 44-month period, 179 consecutive patients underwent DIEP flap breast reconstruction with 242 flaps performed. A retrospective chart review recorded PCA usage, visual analog scale pain scores, and length of stay. **Results:** Pain management with PCA after DIEP flap breast reconstruction was uniformly controlled. Most patients (74.9%) required PCA usage in the first 2 days with conversion to oral analgesics. A subgroup of patients (25.1%) continued to require PCA usage on the third postoperative day. These “nonresponder” patients had a higher visual analog scale score on the first postoperative day, higher total intravenous morphine use, and a longer length of stay (all, *P* < .05). A multivariate analysis revealed more nonresponders among patients undergoing immediate breast reconstruction (*P* < .05); however, all other factors analyzed had no correlation. **Conclusion:** We report a subgroup of patients with poor pain control after DIEP flap breast reconstruction. This group of patients required a longer course of pain management and subsequently a longer hospital stay. Pain management protocols that identify these patients promptly can allow for appropriate modifications.

The proper management of postoperative pain is important in both aesthetic and reconstructive surgery. It has been shown that when pain is well-controlled, outcomes are improved in orthopedic, vascular, general, and cardiac surgery.^1^ Although most patients achieve proper pain control with a standard regimen, some patients require additional attention.^2^ Identification of these patients with poor pain control is necessary in the immediate postoperative period, as an individually tailored pain regimen may be required.

Evaluation of pain management protocols can be confounded by differences in populations chosen for study. Ideally, postoperative pain should be assessed in comparable subjects undergoing similar, if not identical, operations. Previous studies have shown that patients with DIEP flap have significantly less morphine use and shorter hospitalizations than those with musculocutaneous flap for breast reconstruction.^3^,^4^ For patients undergoing DIEP flap breast reconstruction at our institution, a standardized pathway has been developed. The postoperative pain regimen includes patient-controlled analgesia (PCA) for the first 3 days, with conversion to oral narcotics on the morning of the third postoperative day (ie, POD 3). This regimen was developed with the intention of discharging patients as early as the third day. All patients underwent the same standardized activity level with transfer to a chair on the first day and ambulation on the second day. Even with this standardization, there are distinct differences in pain control among different patients and a small group of poorly controlled patients required additional narcotics.

The acute pain literature describes a population of patients that have a poor response to narcotic use in both cancer and postsurgical care. These patients represent 25% to 40% of the population and have been previously described as “nonresponders.”^5^,^6^ These patients experience inadequate pain relief or require doses of analgesics that exceed the standard protocols. Review of the plastic surgery literature revealed few studies examining postoperative pain in a uniform population.

We evaluated narcotic use and postoperative pain control in patients with DIEP flap breast reconstruction within a standardized pathway. We used the visual analog score (VAS) score to assess postoperative pain. The VAS score has been shown to reliably assess acute pain and changes in pain intensity.^7^ The goal was to determine the existence of a nonresponder group and evaluate the characteristics of this subpopulation.

## PATIENTS AND METHODS

Over a 44-month period, all patients who underwent autologous breast reconstruction were identified. All patients had a DIEP flap reconstruction, as superficial inferior epigastric artery (SIEA), free transverse rectus abdominis musculocutaneous (TRAM), muscle-sparing free TRAM, and pedicled TRAM flaps were excluded. Details of the operative technique were standardized including the internal mammary recipient vessel site as well as the abdominal closure; no patients in this series required abdominal mesh placement.

A retrospective chart review of the clinical course, postoperative PCA usage, VAS pain scores, and length of stay was performed. The VAS score was recorded as a daily average. Patients were excluded if there was a return to the operating room, as this altered the standardized pain control regimen. Patients were evaluated the morning of the POD 3 for conversion to oral narcotics. Criteria for conversion to an oral regimen were based on a low overnight VAS score with minimal overnight PCA morphine usage.

A detailed database was reviewed, and data collection included patient demographics, body mass index (BMI), Breast Cancer susceptibility gene (BRCA) status, previous radiation, previous chemotherapy, immediate/delayed reconstruction, unilateral/bilateral, flap weight, mastectomy weight, recipient vessel, number of perforators, and microvascular flap complications.

Patients were categorized as responders if they were successfully weaned from PCA opioids to oral pain medications by the POD 3. Comparisons of intravenous (IV) morphine requirements, VAS scores, and length of stay were analyzed by using the Mann-Whitney *U* test. Comparisons of incidences were performed using a Fisher exact test. Univariate analysis of factors associated with nonresponders was performed; all factors that were found to be significant to the *P* ≤ .25 level were included in multiple logistic regression analyses. Multiple logistic regression with nonresponder status as the dependent variable was performed using backwards stepwise elimination with elimination significance set to .10. Statistical significance was determined at *P* ≤ .05.

The study was approved by the institutional review board at Beth Israel Deaconess Medical Center.

## RESULTS

Over a 44-month period, 179 consecutive patients underwent DIEP flap breast reconstruction with a total of 242 flaps performed. All patients received PCA in the first 3 PODs: 133 patients received morphine and 46 patients received hydromorphone. The hydromorphone dose was converted to morphine with a 5-to-1 conversion.^8^

**Figure 1:**
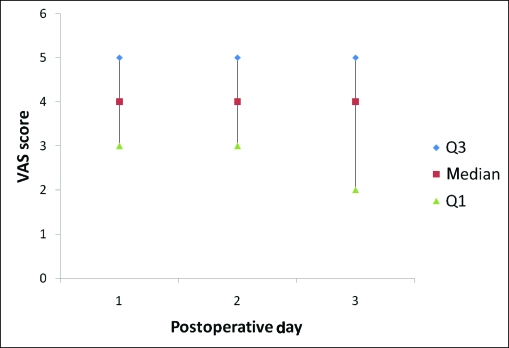
Daily median visual analog scale (VAS) scores for the study population in the first 3 postoperative days (n = 179).

**Figure 2:**
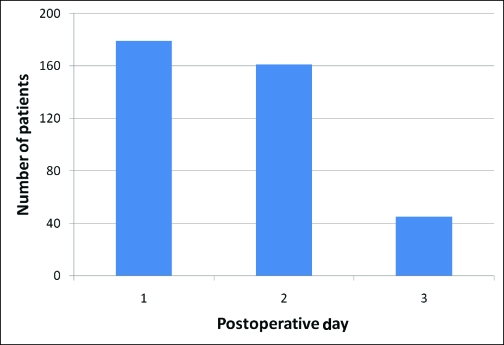
Number of patients requiring PCA for each POD. Patients requiring PCA on POD 3 were identified as nonresponders (45/179, 25.1%). PCA indicates patient-controlled analgesia; POD, postoperative day.

**Figure 3:**
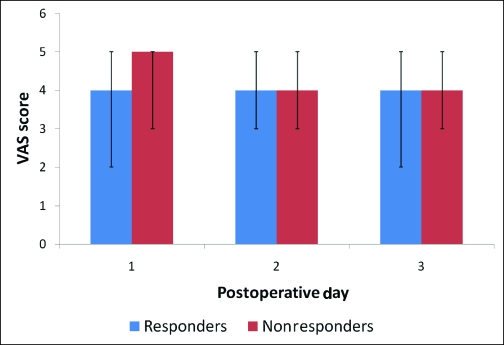
A comparison of average visual analog scale (VAS) scores on each postoperative day between responder (blue) and nonresponder (red) groups. Nonresponders were more likely to have a VAS score 5 or more on the first postoperative day (POD 1) than responders (5 vs 4, *P* < .05).

**Figure 4:**
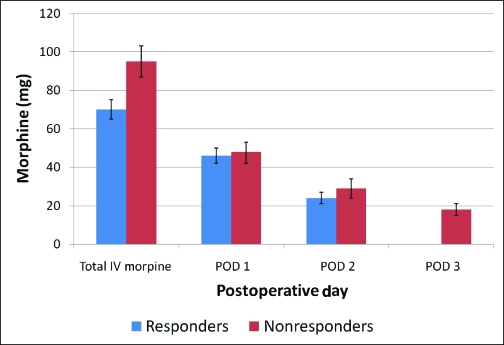
A comparison of daily morphine requirement between responder (blue) and nonresponder (red) groups. Nonresponders had a significantly higher total intravenous (IV) morphine consumption (95 ± 70 mg vs 70 ± 50 mg, *P* < .05).

The median VAS scores during the first 3 PODs revealed that pain was well-controlled for the population as a whole (Fig 1). When analyzing the PCA usage over the first 3 PODs (Fig 2), most patients (134, 74.8%) required PCA usage only in the first 2 days and were quickly converted to oral pain medication. However, a small group of patients (45, 25.1%) was identified, which continued to require PCA usage on the POD 3. This group of nonresponders had a significantly higher VAS score on POD 1 (5 vs 4 *p* < 0.05) (Fig 3). In addition, this population had a significantly higher total IV morphine consumption (95 ± 70 mg vs 70 ± 50 mg, *P* < .05) (Fig 4) and a longer length of stay (4.75 vs 4.2 days, *P* < .05) (Table 1) than the responders group.

**Figure 5:**
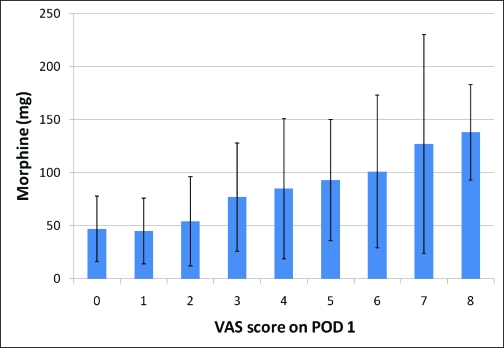
Visual analog scale (VAS) score on the first postoperative day (POD 1) relative to the total intravenous morphine consumption over the entire hospitalization.

We also found a relationship between the VAS score on POD 1 and the total IV morphine consumption (Fig 5). A higher VAS score on POD 1 was associated with a higher total PCA morphine use for the entire hospitalization. Nonresponders were more likely to have a VAS score of five or more on POD 1 than responders (40% vs. 20%; *P* < 0.05). This resulted in a positive predictive value of 39% and a negative predictive value of 81%.

A multivariate logistic regression analysis was performed identifying factors that might influence postoperative analgesia requirements. There was a statistically higher percentage of patients undergoing immediate reconstruction in the nonresponders group compared to delayed reconstruction (29.5% [36/122] vs 15.8% [9/57], *P* < .05). There were no other correlations for age, BMI, BRCA status, a previous history of radiation or chemotherapy, mastectomy weight, flap weight, and unilateral versus bilateral reconstruction (Table 1). There was no correlation for number of perforators used as well.

## DISCUSSION

The DIEP flap breast reconstruction population represents a uniform group of patients amenable to specific evaluation of postoperative pain. With the standardization of pain treatment, identification of factors contributing to pain control can be extracted. Analysis of the data demonstrated that on the POD 3 the majority of patients no longer required PCA. On the basis of VAS scores, these patients, the “responder” group, had a normal response to narcotics with well-controlled pain. This reinforced the efficacy of our postoperative pathway for DIEP flap breast reconstruction—most patients quickly converted to oral narcotics and were discharged home uneventfully.

We found a distinct nonresponder subgroup of patients who had elevated pain scores despite higher PCA consumption. This group was characterized by a significantly higher VAS score on the POD 1, a greater total IV morphine consumption, and a longer hospital stay. In addition, their VAS score and daily morphine usage were higher than those of the responder group. Multivariate analysis revealed that a higher percentage of these patients had an immediate breast reconstruction.

Stamer et al^5^ were the first to identify a subgroup of nonresponders who had higher pain scores and required more analgesic consumption than their “normal” counterparts. Other studies have suggested that variance in postoperative pain control could result from differential pain receptor densities among patients. Patients typically demand more analgesia from a PCA, as their plasma concentration drops below a minimum effective concentration.^9^ This concentration shows considerable variation between patients, suggesting that there may be constitutional sensitivity differences at the level of the opioid receptors. The µ-opioid peptide receptor (MOP) has been identified as the principal site for pharmacologic action of most clinically important opiate drugs. Recent studies using various knockout mice and recombinant-inbred strain CXBK mice models have indicated that the analgesic effect of morphine is directly dependent on MOP densities.^10^ There are more than 100 polymorphisms identified in the human MOP (*OPRM1*) gene which could explain the interindividual differences in pain control by opiate analgesia.^10^

Stamer et al^5^ reported a higher rate of nonresponders (40%) than our study group (25.2%). This could be attributed to differences in study design, as they evaluated patients undergoing multiple types of operations. Their nonresponder group included a higher proportion of patients undergoing major abdominal surgery than the responders group. In our study design, a homogeneous patient population was identified. Both groups were similar in operative detail and extent (number of perforators, unilateral versus bilateral, mastectomy weight and flap weight). Our percentage of nonresponders is closer to the known variability described in cancer patients. The acute pain literature describes a nonresponder population in patients treated with narcotics for pain related to cancer. In this population, 20% to 34% of patients had a clinical failure to narcotics requiring a change in pain management.^6^,^11^

Our study revealed that a nonresponder could be predicted on the POD 1 by a significantly higher VAS score. Previous studies were able to identify 89.2% of the nonresponders based on the size of the loading dose and the pain scores in the first 30 minutes after surgery.^5^ Others studies have also shown the importance of the loading dose in predicting postoperative analgesia requirements. Butscher et al^12^ used the initial IV bolus dose to predict the need for intermittent intramuscular injections after surgery. Macintyre and Jarvis showed in 78 postoperative patients that pain scores and loading dose correlated with morphine consumption during the first 24 hours.^13^

The ability to predict the nonresponder patients in the POD 1 is extremely important. As the nonresponder group consumes higher total morphine amounts, they experience poor pain control at the same time. Reducing the opioid dose has been shown to decrease the opioid-related side effects (nausea, dizziness, fatigue, sleep disturbance, and ileus).^14^ Improvements in pain control combined with a reduction in side effects can directly affect the length of stay. We found this to be significant as the length of stay was longer in our nonresponder group (4.75 vs 4.2 days, *P* < .05).

With early detection of nonresponders, we can potentially improve postoperative pain management by combining different types of systemic analgesics. The major benefit of multimodal analgesia may be a decrease in the incidence of opioid side effects rather than an increase in the effectiveness of pain control.^1^ In addition, early intervention may potentially decrease length of stay. The addition of gabapentin in the postoperative pain regimen has been shown to reduce postoperative narcotic use, and further studies evaluating efficacy in this subgroup are underway.^15^ Finally, the use of continuous local anesthetic infusion pain pumps has been described for use in breast reconstruction recently and may represent another modality for pain control in this population.^16^^-^19

Our study has several limitations. As our postoperative pain data was collected using IV PCA, it is not clear how pain is comparably controlled on oral pain medications. It would be important to determine whether a similar nonresponder group exists for oral opioids. With the shift of many plastic surgery procedures to an outpatient setting, identification and intervention in this subset of patients could potentially improve postoperative care on a wider scale. Another limitation is the psychological response to postoperative pain. As there are emotional and learned responses to pain, further collaborations are necessary to evaluate these associated psychological components.

The last limitation is that there are minute technical differences even among DIEP flap reconstruction patients. As the amount of muscle dissection and nerve damage can be variable based on the anatomy, this can affect postoperative pain. Our study did not find associations between number of perforators used and pain; however, nerve damage and injury can be difficult to quantify and can certainly contribute to postoperative pain.

The presence of a nonresponder group is important in any postsurgical setting. As opioid receptor characteristics are universal, recognition of this subgroup is critical and not confined to one type of surgery.^5^ Postoperative pain is relevant in all aspects of plastic surgery from aesthetic to reconstructive surgery. Proper evaluation of different uniform postoperative populations is necessary to correlate with our findings. If nonresponders can be detected as early as the first postoperative hour, their pain regimen can be adjusted accordingly and may improve their postoperative course.^5^ More precise studies on the relationship between gene polymorphisms and opioid sensitivity can one day individualize unique pain management protocols for every patient.

## CONCLUSION

Our study identifies a group of patients who have a poor response to standardized pain control regimens after a DIEP flap breast reconstruction. This group of nonresponders requires more IV PCA morphine and has a longer length of stay. We can identify these patients on the basis of the VAS score on the POD 1 and can potentially alter their pain management and improve patient outcomes. We hope to design future prospective trials to address this group.

## Figures and Tables

**Table 1:** Comparison of Responder and Nonresponder Groups

	Responders (n = 134)	Nonresponders (n = 45)	*P*
Patient characteristics			
Age, mean ± SD, y	47.5 ± 8.0	47.9 ± 7.8	.78
Mastectomy weight, mean ± SD, g	696 ± 371	654 ± 309	.56
Flap weight, mean ± SD, g	652 ± 264	679 ± 207	.48
BRCA genetic mutation	13% (n = 18)	13% (n = 6)	1.0
Radiation therapy	26% (n = 35)	24% (n = 11)	1.0
Chemotherapy	19% (n = 26)	11% (n = 5)	.26
Bilateral	34% (n = 46)	40% (n = 18)	.59
Delayed	64% (n = 86)	80% (n = 36)	.64
Body Mass Index, mean ± SD, kg/m^2^	26.5 ± 5.0	27.0 ± 5.0	.54
Length of stay, d	4.2	4.75	*P* < .05*
	Postoperative pain characteristics		
Visual analog scale score	4/10 (2-5)	5/10 (3-5)	*P* < .05*
Total intravenous morphine, mg	70 ± 50	95 ± 70	*P* < .05*

* Visual analog scale score represents the mean score during the first 3 days. Total intravenous morphine represents the total morphine usage over the first 3 days. Mann-Whitney *U* test for statistical analysis.
